# Supplementary material for: Prevalence of clinically significant refractive error in children in Europe: Systematic review and meta-analysis
Source: PLoS One. 2025 Nov 12;20(11):e0335666. doi: 10.1371/journal.pone.0335666 (PMC12611104; doi:10.1371/journal.pone.0335666)
Supplement: S1 Appendix — (PDF) [file pone.0335666.s001.pdf]

The Prevalence of Refractive Error in the Paediatric Population of Europe

Review Question

The primary question this review aims to answer is "What is the prevalence of refractive error in the paediatric population of Europe?" Meta-analysis will be employed to evaluate the prevalence estimate in this cohort of the population. Data will be sourced through a systemic search of the literature, collated, and analysed. Refractive error will include myopia, hyperopia, and astigmatism with additional categories for higher levels of both hyperopia and myopia. Anisometropia prevalence will also be included as an additional objective. All paediatric populations within Europe will be included with distinctions made between countries for data analysis purposes. Age groups from 0 to 16 will be included permitting refractive error has been assessed in intervals of 5 years or less. All genders will be included with division into male and female subgroups as appropriate. Subgroups of both rural and urban populations will also be included.

Secondary review questions that may be answered depending on the available literature include:
"What is the prevalence of anisometropia in the paediatric population of Europe?'
"How does refractive error prevalence vary between different regions in Europe?"
"How does refractive error prevalence vary according to age?"
"How does refractive error prevalence levels vary depending on gender?"
"How does refractive error prevalence levels vary depending on levels of urbanisation?"

These secondary questions will facilitate further understanding of the distribution of refractive error throughout Europe and among populations as well as the prevalence of anisometropia.

Searches

PubMed
Cochrane

Types of study to be included

Studies that investigate one or more types of refractive error and/or anisometropia prevalence in paediatric populations within Europe

Inclusion Criteria

- Studies with clear definitions and thresholds of refractive error
- Studies that specifically report sample sizes
- Studies that outline the use (or not) of cycloplegic agents
- Studies that quantify prevalence of refractive error
- Studies examining and that are representative of European paediatric populations

Exclusion Criteria

- Studies published prior to the year 2000
- Studies with participants greater than 16 years old
- Studies that assess refractive error levels in greater than 5-year age intervals

- Self-reported refractive error
- Studies that do not distinguish between refractive error sub-types
- Studies that define refractive error in terms of ocular pathology such as astigmatism levels in patients with keratoconus
- Studies that are not representative of the population such as those defined by disease

Condition or domain being studied

The prevalence of refractive error and anisometropia in human children within Europe. Refractive errors include myopia (short-sightedness), hyperopia (far-sightedness) and astigmatism (distorted vision due to the shape of the front of the eye and/or lens). Higher levels of both myopia and hyperopia will also be assessed in this study. Anisometropia refers to significant intra-ocular difference in refractive error. Further analysis based on age group, gender, specific region, and urbanisation.

Participants/population

This review aims to representative of the paediatric population in Europe. It includes studies of human populations, with participants between 0-16 years residing in the European countries (as defined by the World Health Organisation) of Albania, Andorra, Armenia, Austria, Azerbaijan, Belarus, Belgium, Bosnia and Herzegovina, Bulgaria, Croatia, Cyprus, Czechia, Denmark, Estonia, Finland, France, Georgia, Germany, Greece, Hungary, Iceland, Ireland, Italy, Kazakhstan, Kyrgyzstan, Latvia, Lithuania, Luxembourg, Malta, Monaco, Montenegro, Netherlands, North Macedonia, Norway, Poland, Portugal, Republic of Moldova, Romania, Russian Federation, San Marino, Serbia, Slovakia, Slovenia, Spain, Sweden, Switzerland, Tajikstan, Turkey, Turkmenistan, Ukraine, United Kingdom of Great Britain and Northern Ireland and Uzbekistan.

Intervention(s), exposure(s)

There will be no interventions directly assessed in this review, however, refractive error and anisometropia are products of both genetic and environmental influences therefore exposure may be indirectly examined as a result. Interventional studies will inherently be excluded from this review as it is specifically focused on prevalence levels. Countries with public health interventions which may influence refractive error such as the use of myopia control interventions will not be explicitly excluded. The epidemiological nature of this research lends itself to the evaluation of geographical and time related trends in refractive error prevalence.

Comparator(s)/control

The requirement for a control is not indicated due the epidemiological nature and meta-analysis structure of this research.

Context

This review will only examine prevalence levels in the context of the continent of Europe and the paediatric sub-group of this population.

Main outcome(s)

Estimation of the prevalence of refractive error in the paediatric population of Europe. Specifically, outcomes will be sought regarding the prevalence of the following refractive errors within the paediatric population in Europe (in no order):

1. Myopia
2. High Myopia
3. Hyperopia
4. High Hyperopia
5. Astigmatism

All outcomes will be equally prioritised.

Additional outcome(s)

Assessment of the prevalence of anisometropia in addition to the distribution of refractive error prevalence based on region, sex, age, and urbanisation. The impact of refractive error definitions and the influence of the use of cycloplegic agents in determining refractive error prevalence may also be assessed if possible.

Data extraction (selection and coding)

Two researchers will search the databases for suitable studies. Data will be selected based on fulfilment of the eligibility criteria. At this stage two independent reviewers will select eligible studies by reviewing their content for suitability. Conflicts will be resolved through discussion and the input of third researcher who will act as a mediator. This applies to both the screening and eligibility phases of the review. Two researchers will collate the data using Excel Spreadsheet. Data will be extracted from studies by reviewing the methods and result sections. Data will be collected in the following categories:

1. Year of Study
2. Country of Study
3. Region of Study
4. Urban/Rural Setting of Study if applicable
5. Age Group of Study
6. Gender Studied
7. Use of Cycloplegic Agents
8. Levels of Myopia (as percentage of population studied)
9. Study Definition of Myopia
10. Levels of High Myopia (as percentage of population studied)
11. Study Definition of High Myopia
12. Levels of Hyperopia (as percentage of population studied)
13. Study Definition of Hyperopia
14. Levels of High Hyperopia (as percentage of population studied)
15. Study Definition of High Hyperopia
16. Levels of Astigmatism (as percentage of population studied)
17. Study Definition of Astigmatism
18. Levels of Anisometropia (as percentage of population studied)

19. Study Definition of Anisometropia

Investigators will be contacted for confirmation of data as required. There are no pre-planned assumptions or simplifications to report at this stage of the review. Data will be analysed by a fourth independent researcher using the computer programming software 'R.' Any duplicate data will be removed.

Risk of bias (quality) assessment

Methodological quality of included studies will be evaluated using the tool developed by the Joanna Briggs Institute.

Strategy for data synthesis

The pooled prevalence of refractive errors, myopia, high myopia, hyperopia, high hyperopia, astigmatism and anisometropia will be provided with 95% confidence intervals. The I^2 test will be performed to estimate the heterogeneity of the included studies. Egger's tests will be used to access the publication bias of the studies. A statistically significant publication bias is considered if the p value ≤ 0.05.

Analysis of subgroups or subsets

Age subgroups – age groups from 0 to 16 years in 5-year intervals
Sex - male and female subgroups
Ethnicity – all subgroups will be considered but due to the area under study, it is likely the population will represent ethnicities common in Europe
Urbanisation - "rural" and "urban" subgroups

Contact details for further information

Aoife O Donnell

aoifeodonnell@outlook.ie

Organisational affiliation of the review

School of Physics, Clinical and Optometric Sciences

Review team members and their organisational affiliations

Ms. Aoife O Donnell. School of Medicine, Ulster University

Dr. Michael Moore. School of Physics, Clinical and Optometric Sciences, Technological University Dublin.

Ms. Megan Doyle. School of Physics, Clinical and Optometric Sciences, Technological University Dublin.

Dr. Siofra Harrington. School of Physics, Clinical and Optometric Sciences, Technological University Dublin.

Dr. Veronica O'Dwyer. School of Physics, Clinical and Optometric Sciences, Technological University Dublin.

Type and method of review

Meta-analysis, Systematic review, Epidemiologic.

Anticipated or actual start date

28th March 2022

Anticipated completion date

31st July 2023

Funding sources/sponsors

There are no sources of funding or sponsors to declare.

Conflicts of interest

There are no known conflicts of interest.

Language

English

Country

Ireland

Stage of review

Ongoing
