# Supplementary figures and images for: Prevalence of clinically significant refractive error in children in Europe: Systematic review and meta-analysis
Source: PLoS One. 2025 Nov 12;20(11):e0335666. doi: 10.1371/journal.pone.0335666 (PMC12611104; doi:10.1371/journal.pone.0335666)

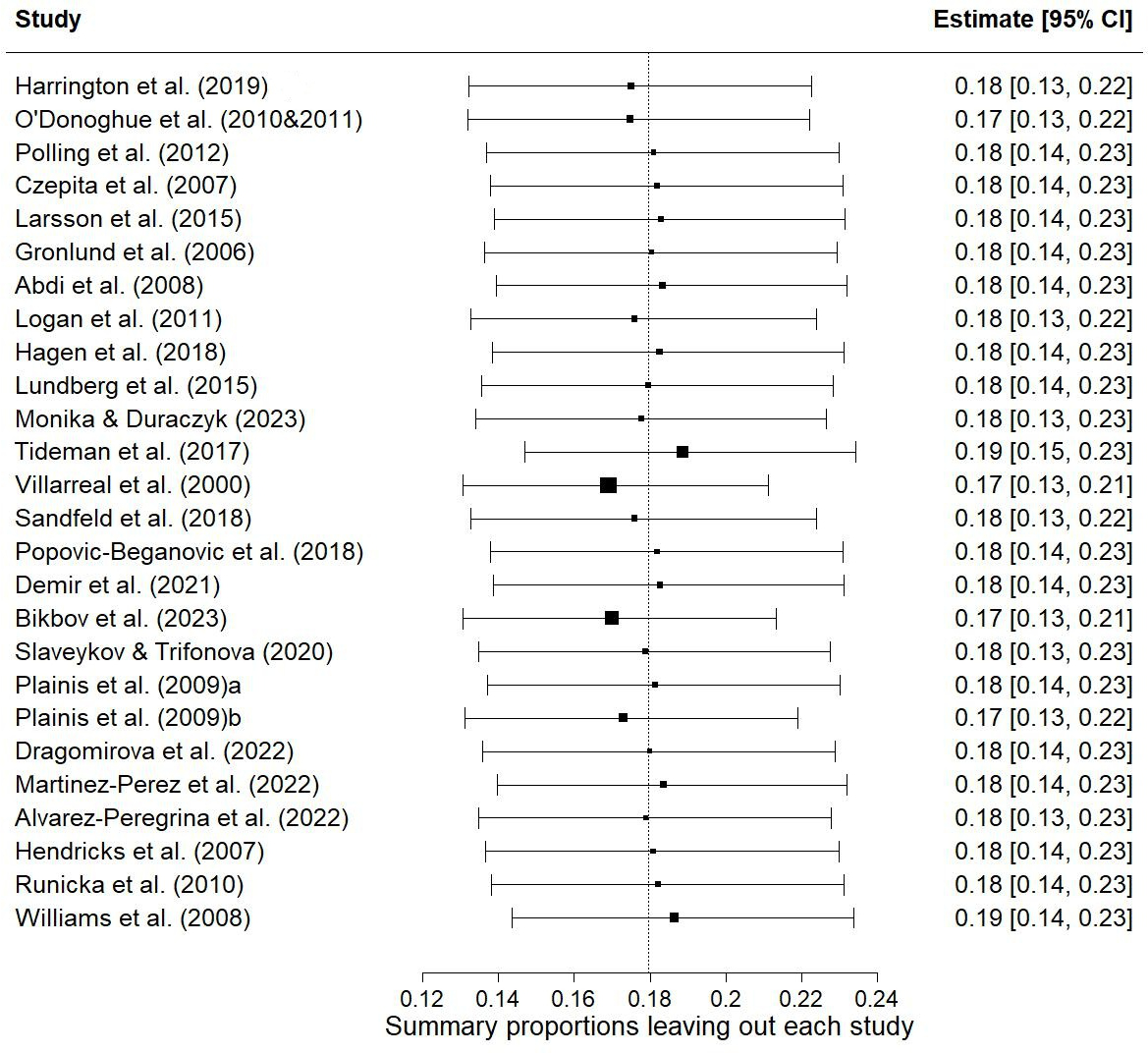

Supplement: S1 Fig — Leave-one-out analyses were performed to determine how much individual studies affect the pooled estimates of the other studies. (TIF) [file pone.0335666.s002.tif]

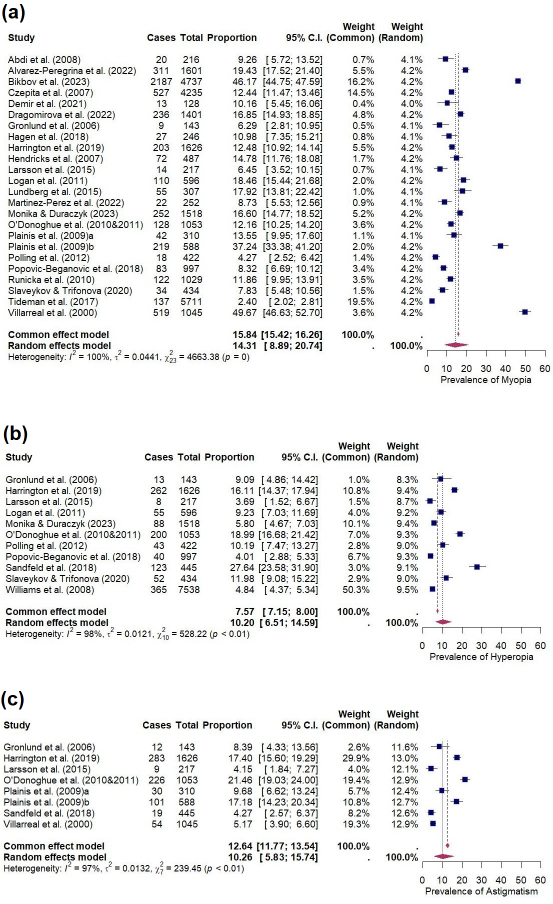

Supplement: S2 Fig — Myopia prevalence was 14.31% (95% CI: 8.89–20.74). Hyperopia prevalence was 10.20% (95% CI: 6.51–14.59). Astigmatism prevalence was 10.26% (95% CI: 5.83–15.74). (TIF) [file pone.0335666.s003.tif]

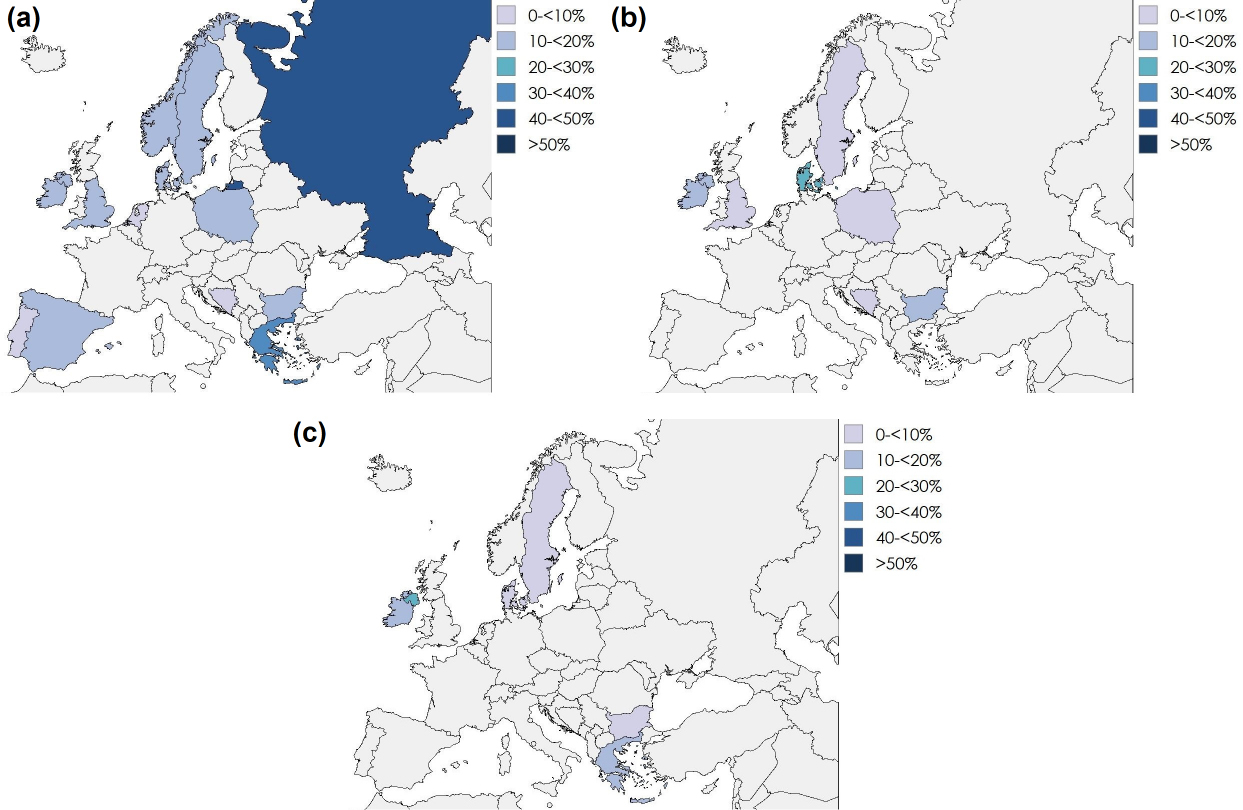

Supplement: S3 Fig — Figure 3(a) depicts myopia (≤−0.50D) prevalence, (b) hyperopia (≤+2.00D) prevalence and (c) astigmatism (≥1.00 DC) prevalence. Mapping data from http://www.naturalearthdata.com/. (TIF) [file pone.0335666.s004.tif]
