# Supplementary material for: Prevalence of clinically significant refractive error in children in Europe: Systematic review and meta-analysis
Source: PLoS One. 2025 Nov 12;20(11):e0335666. doi: 10.1371/journal.pone.0335666 (PMC12611104; doi:10.1371/journal.pone.0335666)
Supplement: S3 Table — (DOCX) [file pone.0335666.s007.docx]

Supplemental Table 3. Critical Appraisal of Included Papers Using the Joanna Briggs Institute tool.

| Year | Study | Item 1 | Item 2 | Item 3 | Item 4 | Item 5 | Item 6 | Item 7 | Item 8 | Item 9 | Score |
| --- | --- | --- | --- | --- | --- | --- | --- | --- | --- | --- | --- |
| 2000 | Villarreal et al | Yes | Yes | Unclear | Yes | Yes | Yes | Yes | Yes | Yes | 8 |
| 2006 | Grönlund et al | Yes | Yes | Unclear | Yes | Yes | Yes | Yes | Yes | Unclear | 7 |
| 2007 | Czepita et al | Yes | Yes | Yes | Yes | Yes | Yes | Yes | Yes | Yes | 9 |
| 2007 | Hendricks et al | Yes | Unclear | Unclear | Yes | Yes | Yes | No | Yes | Yes | 6 |
| 2008 | Abdi et al | Yes | Yes | Yes | No | Yes | Yes | No | Unclear | Yes | 6 |
| 2008 | Williams et al | Yes | Yes | Yes | Yes | Yes | Yes | Yes | Yes | Yes | 9 |
| 2009 | Plainis et al | Yes | Unclear | Unclear | Yes | Yes | Yes | Yes | Yes | Yes | 7 |
| 2010 | O'Donoghue et al | Yes | Yes | Yes | Yes | Yes | Yes | Yes | Yes | Yes | 9 |
| 2010 | Rudnicka et al | Yes | Yes | Yes | Yes | Yes | Yes | Yes | Yes | Yes | 9 |
| 2011 | Logan et al | Yes | Yes | Yes | Yes | No | Yes | Yes | Yes | Unclear | 7 |
| 2011 | O'Donoghue et al | Yes | Yes | Yes | Yes | Yes | Yes | Yes | Yes | Yes | 9 |
| 2012 | Polling et al | Yes | Yes | Yes | Yes | Yes | Yes | Yes | Yes | Yes | 9 |
| 2015 | Larsson et al | Yes | Yes | Unclear | Yes | No | Yes | Yes | Yes | Yes | 7 |
| 2015 | Lundberg et al | Yes | Yes | Unclear | Yes | Yes | Yes | Yes | Yes | Yes | 8 |
| 2015 | O’Donoghue et al | Yes | Yes | Yes | Yes | Yes | Yes | Yes | Yes | Yes | 9 |
| 2016 | McCullough et al | Yes | Yes | Yes | Yes | Yes | Yes | Yes | Yes | Yes | 9 |
| 2017 | Tideman et al | Yes | Yes | Yes | Yes | Yes | Yes | Yes | Yes | Yes | 9 |
| 2018 | Popović-Beganović et al | Yes | Yes | Yes | Yes | Yes | Yes | Yes | Yes | Yes | 9 |
| 2018 | Hagen et al | Yes | Yes | No | Yes | No | Yes | Yes | Yes | No | 6 |
| 2018 | Sandfeld et al | Yes | Yes | Unclear | Yes | Yes | Yes | Yes | Yes | Yes | 8 |
| 2019 | Harrington et al | Yes | Yes | Yes | Yes | Yes | Yes | Yes | Yes | Yes | 9 |
| 2020 | Slaveykov & Trifonova | Yes | Yes | Unclear | Yes | Yes | Yes | Yes | Yes | Unclear | 7 |
| 2021 | Demir et al | Yes | No | Yes | Yes | Yes | Yes | Yes | Yes | Unclear | 7 |
| 2021 | Alvarez-Peregrina et al | Yes | Yes | Unclear | Unclear | Yes | Yes | Yes | Yes | Unclear | 6 |
| 2022 | Dragomirova et al | Yes | Unclear | Unclear | Yes | Yes | Yes | Yes | Yes | Unclear | 6 |
| 2022 | Martinez-Perez et al | Yes | Unclear | Unclear | Yes | Yes | Yes | Yes | Yes | Unclear | 6 |
| 2023 | Monika et al | Yes | Unclear | Unclear | Yes | Yes | Yes | Yes | Yes | Unclear | 6 |
| 2023 | Bikbov et al | Yes | Yes | Unclear | Yes | Yes | Yes | Yes | Yes | Unclear | 7 |

*Item 1: Was the sample frame appropriate to address the target population? Item 2: Were study participants sampled in an appropriate way? Item 3: Was the sample size adequate? Item 4: Were the study subjects and setting described in detail? Item 5: Was the data analysis conducted with sufficient coverage of the identified sample? Item 6: Were valid methods used for the identification of the condition? Item 7: Was the condition measured in a standard, reliable way for all participants? Item 8: Was there appropriate statistical analysis? Item 9: Was the response rate adequate, and if not, was the low response rate managed appropriately?*
