# Supplementary material for: Prevalence of clinically significant refractive error in children in Europe: Systematic review and meta-analysis
Source: PLoS One. 2025 Nov 12;20(11):e0335666. doi: 10.1371/journal.pone.0335666 (PMC12611104; doi:10.1371/journal.pone.0335666)
Supplement: S4 Table — (DOCX) [file pone.0335666.s008.docx]

Supplemental Table 4. Grading of Recommendations, Assessment, Development and Evaluation (GRADE) Summary of Findings.

| **Prevalence of Clinically Significant Refractive Error in Children in Europe and the Need for Standardised Reporting: A Systematic Review and Meta-Analysis** |
| --- |
| **Population:** Children aged 4-17 years in Europe  **Outcome:** Refractive Error Prevalence  **Study Design:** Observational Studies |
| **The pool refractive error prevalence** of 4-17-year-old children (and its 95% confidence interval) is based on the included 27 observational studies with 37,282 participants in terms of cycloplegia status, age and sex. |
| **GRADE Working Group grades of evidence** (Balshem *et al.*, 2011).  **High certainty:** very confidence that the true effect lies close to that of the estimate of the effect.  **Moderate certainty:** moderately confident that the true effect is likely to be close to the estimate of the effect, but there is a possibility that it is substantially different.  **Low certainty:** limited confidence in the effect estimate – the true effect may be substantially different.  **Very low certainty:** very little confidence in the effect estimate – the true effect is likely to be substantially different. |
| **Explanations**   1. Overall risk of bias is serious. Limitations in geographical coverage, older age groups, less reporting on hyperopia and astigmatism prevalence, differing definitions of hyperopia and astigmatism. 2. I-squared value of 100%. |
