# Supplementary material for: Prevalence of clinically significant refractive error in children in Europe: Systematic review and meta-analysis
Source: PLoS One. 2025 Nov 12;20(11):e0335666. doi: 10.1371/journal.pone.0335666 (PMC12611104; doi:10.1371/journal.pone.0335666)
Supplement: S5 Table — (DOCX) [file pone.0335666.s009.docx]

Supplemental Table 5. Excluded papers and reason for exclusion.

|  | First Author (year) | Reason for Exclusion |
| --- | --- | --- |
| 1 | Vodencarevic (2020) | Only included participants who failed screening |
| 2 | Jrbashyan (2022) | Only included participants who failed screening |
| 3 | Falkenberg (2019) | Only included participants who failed screening |
| 4 | Bruce (2018) | Only included participants who failed screening |
| 5 | Georgelin (2021) | Only included refractive error category breakdown for participants who failed screening |
| 6 | Truckenbrod (2021) | Refractive error definitions do not conform to review |
| 7 | Semeraro (2020) | Participants too young for review inclusion |
| 8 | Atkinson (2007) | Refractive error definitions do not conform to review |
| 9 | Anker (2003) | Refractive error definitions do not conform to review |
| 10 | Vojnikovic (2013) | No refractive error definitions |
| 11 | Ore (2014) | No refractive error definitions |
| 12 | Ciumbaraite (2017) | No refractive error definitions |
| 13 | Majauskiene (2005) | No refractive error definitions |
| 14 | Nunes (2022) | No refractive error definitions |
| 15 | Bro (2023) | No refractive error definitions |
| 16 | Toufeeq (2014) | No refractive error definitions |
| 17 | Shah (2017) | No refractive error definitions |
| 18 | Lanza et al (2023) | No refractive error definitions |
| 19 | Szaflik (2004) | No refractive error definitions |
| 20 | Mukazhanova (2022) | Population not representative of European population |
| 21 | Caca (2013) | Population not representative of European population |
| 22 | Gursoy (2013) | Population not representative of European population |
| 23 | Akova-Budak (2015) | Population not representative of European population |
| 24 | Parssinen (2017) | Only myopes included – not representative of general population |
| 25 | Prsova (2023) | Sample too small to be representative |
| 26 | Caputo (2001) | Not representative of general population |
| 27 | Armarnik (2021) | Not representative of general population |
| 28 | Battagliola (2023) | Not representative of general population (clinic-based) |
| 29 | Reimelt (2018) | Not representative of general population |
| 30 | Rayapoulle (2021) | Refractive error reported by survey |
| 31 | Mandalos (2002) | Refractive error reported by survey |
| 32 | Mavrakanas (2000) | Refractive error reported by survey |
| 33 | Jobke (2008) | Refractive results from records – no information on cycloplegia status |
| 34 | Nemeth (2013) | Does not differentiate refractive error prevalence between children and adult participants |
| 35 | Guggenheim et al (2012) | Duplicate of ALSPAC data (Williams et al 2008) |
| 36 | Sanchez-Tena (2024) | Duplicate of Alvarez-Peregrina (2022) data |
| 37 | Little (2014) | Duplicate of NICER data (O’Donoghue 2010, 2011) |
| 39 | Breslin (2013) | Duplicate of NICER data (O’Donoghue 2010, 2011) |
| 40 | Enthoven (2020) | Duplicate of Generation R data (Tideman 2017) |
| 41 | Enthoven (2021) | Duplicate of Generation R data (Tideman 2017) |
| 42 | Harrington (2019) | Duplicate of IES data (Harrington 2019) |
