# Supplementary material for: Prevalence of clinically significant refractive error in children in Europe: Systematic review and meta-analysis
Source: PLoS One. 2025 Nov 12;20(11):e0335666. doi: 10.1371/journal.pone.0335666 (PMC12611104; doi:10.1371/journal.pone.0335666)
Supplement: Attachment — Submitted filename: Letter of Response to Reviewers.docx [file pone.0335666.s010.docx]

Dr Emily Chenette, PhD
Editor-In-Chief
PLoS One
11^th^ September 2025

Dear Doctor Chenette,

Thank you for the opportunity to submit our revised manuscript entitled "Prevalence of Clinically Significant Refractive Error in Children in Europe: Systematic Review and Meta-Analysis" for consideration for publication in PLoS One.

The authors thought the feedback was positive, with helpful pointers for the paper. The authors appreciate the time and effort the reviewers have dedicated to providing valuable feedback on the manuscript. The authors have been able to incorporate changes to reflect the suggestions provided by the reviewers.

In summary, having considered the comments in some detail, the following amendments have been made:

1. Two short sections have been added to clarify points highlighted by Reviewer 1.
2. Lines 50-51, and lines 57 have been rephrased as per Reviewer 2 comments.

The authors have highlighted the changes within the manuscript using tracked changes. Here is a point-by-point response, including edits (italicised) to the reviewers' comments and concerns (in boldface).

**Reviewer: 1
Dear Authors,
I have few concerns regarding the data accuracy. You included in your analysis a recent study from Russia (Bikbov et al., 2023) on a very consistent group of subjects. I read the abstract of this study and actually there is no mention in this study about Hyperopia incidence (in your table appears as being 11.1%) because it is a study focused exclusively on Myopia. From the clinical point of view, I think your analysis is expanded on a very large period and the cohorts included in the studies are very heterogeneous and the large groups influence the final statistics.**

The authors thank the reviewer for their thoughtful comments and the opportunity to clarify these points.

With regard to the concern about the Russian study by Bikbov et al.,^1^ while hyperopia prevalence is not mentioned in the abstract, it is reported in the main body of the paper. Specifically, in the results section (page 594), the authors state: “… and the prevalence of hyperopia (>0.50 D) was 524/4737 (11.1%; 95% CI 10.2–12.0%).” The value reported in Table 2 of the current manuscript accurately reflects this finding.

On the issue of study periods, there is currently limited refractive error prevalence data available for children in Europe. Restricting the timeframe would have reduced the evidence included in the current analysis. Previous meta-analyses of myopia prevalence have adopted similar inclusion periods; for example, Liang et al.^2^ analysed studies from 1990-2023, while Hashemi et al.^3^ included data from 1990-2016. The approach used in the current meta-analysis therefore aligns with established practice and extends the evidence base by incorporating refractive error types beyond myopia.

This was clarified in the manuscript as follows:

**2.1 Literature Search Strategy, Lines 87-92:** “*To maximise the available evidence, studies published from 1 January 2000 to 30 September 2024 were included. This broad timeframe was consistent with previous prevalence meta-analyses and ensured the inclusion of recent, methodologically robust studies while allowing adequate data coverage for European children, where studies remain limited.^5,6,10^*”

The authors agree with the reviewer that heterogeneity is an inherent feature of prevalence meta-analyses, reflecting variations in study populations, geography, methodology, sample size, and timeframes.^4^ To address this, a random-effects model was applied, which is designed to account for both within- and between-study variability.^5^ Additionally, Freeman-Tukey double arcsine transformation and leave-one-out sensitivity analyses were employed to reduce the influence of outlying prevalence estimates and to ensure that larger studies did not disproportionately affect the pooled results.^6^ Taken together, these methodological choices follow established best practices and provide robust estimates despite the unavoidable heterogeneity in the available data.

This was clarified in the manuscript as follows:

**4.8 Strengths, Limitations and Future Directions, Lines 445-447:** “*The broad timeframe and heterogeneous cohorts may have influenced the pooled estimates. This is an inherent feature of prevalence meta-analyses, and the use of a random-effects model with sensitivity analyses was intended to account for this.^69-71^*”

References:

1. Bikbov MM, Kazakbaeva GM, Fakhretdinova AA, Tuliakova AM, Iakupova EM, Panda-Jonas S, et al. Prevalence and associated factors of myopia in children and adolescents in Russia: the Ural Children Eye Study. BJO. 2024;108:593-598.
2. Liang J, Pu Y, Chen J, Liu M, Ouyang B, Jin Z, et al. Global prevalence, trend and projection of myopia in children and adolescents from 1990 to 2050: A comprehensive systematic review and meta-analysis. BJO. 2024;109(3):362-71.
3. Hashemi H, Fotouhi A, Yekta A, Pakzad R, Ostadimoghaddam H, Khabazkhoob M. Global and regional estimates of prevalence of refractive errors: Systematic review and meta-analysis. J Curr Ophthalmol. 2018;30:3–22.
4. Migliavaca CB, Stein C, Colpani V, Barker T, Ziegelmann P, Munn Z, et al. Meta-analysis of prevalence: I^2^ statistic and how to deal with heterogeneity. Res Synth Methods. 2022;13(3):363-367.
5. Borenstein M, Hedges LV, Higgins JPT, Rothstein HR. Random-Effects Model. In: *Introduction to Meta-Analysis*. Wiley;2009:69-75.
6. Barendregt JJ, Doi SA, Lee YY, Norman RE, Vos T. Meta-analysis of prevalence. J Epidemiol Community Health. 2013;67:974-978.

**Reviewer: 2
General comments:
The article is well written, with a clearly defined PICO framework and well-articulated aims. The methodology meets the criteria for a meta-analysis, with appropriate use of plots and figures to present the findings. The conclusions are clearly stated and consistent with the results.**

The authors thank the reviewer for their positive feedback.  **Minor comments:
1- Introduction, line 50
Consider specifying “uncorrected refractive error” instead of “refractive error” to be more precise, as the burden on visual impairment is primarily due to uncorrected cases.**

The authors thank the reviewer for this suggestion. The introduction has been updated to reflect this as follows:
**Lines 50-51: “***Uncorrected refractive error is a leading cause of visual impairment worldwide and a growing public health concern, particularly in children.*” **2- Introduction, line 57
The first sentence could be rephrased for clarity. For example: “While severe refractive error carries greater individual risk, the broader public health burden stems from the vast number of people with low to moderate refractive error, particularly myopia.”**

The authors thank the reviewer for their comment. The introduction has been updated to incorporate this as follows:

**Lines 57-59:** “*While severe refractive error carries greater individual risk, the broader public health burden stems from the vast number of people with low to moderate levels of refractive error, particularly myopia.*”

**Editor Comments:**

The manuscript has been updated to follow the style requirements. The competing interests and funding statements have been updated as requested. Figure 4 and S3 have been recreated using public domain data which is cited in the figure legend.

The authors thank the editorial board and the reviewers for their constructive comments. We appreciate your input, which has improved the paper's clarity and readability.

Yours sincerely,

Michael Moore (corresponding author), Centre for Eye Research Ireland, School of Physics, Clinical & Optometric Sciences, Technological University Dublin, City Campus, Grangegorman, Dublin, Ireland. Email: michael.moore@tudublin.ie
